# Supplementary figures and images for: A Comprehensive Toolkit for Quick and Easy Visualization of Marker Proteins, Protein–Protein Interactions and Cell Morphology in Marchantia polymorpha
Source: Front Plant Sci. 2020 Oct 15;11:569194. doi: 10.3389/fpls.2020.569194 (PMC7593560; doi:10.3389/fpls.2020.569194)

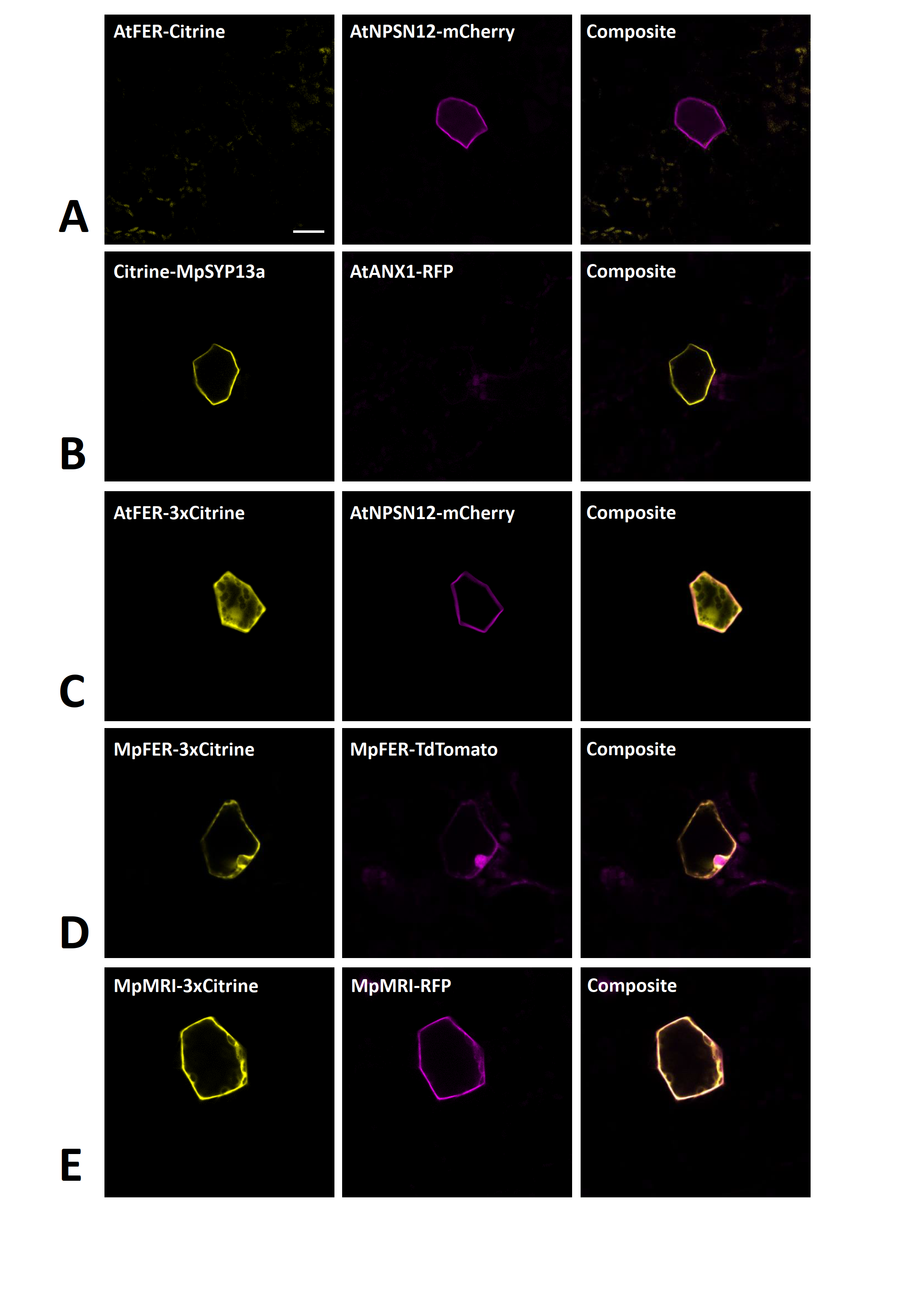

Supplement: Supplementary Figure 1 — Co-expression of MLRs and MRI with single or triple tags in Marchantia epidermal cells. (A,B) Arabidopsis MLRs fused to single fluorescent tag are not expressed. (C,D) The 3xCitrine tag leads MLRs to localize to the cytoplasm. (E) The 3xCitrine tag leads to normal cytosolic and plasma membrane localization of MpMRI. Pictures show maximum projections of z-stack captions (see “Materials and Methods” section for details). Scale bar = 20 μm. [file Image_1.TIF]

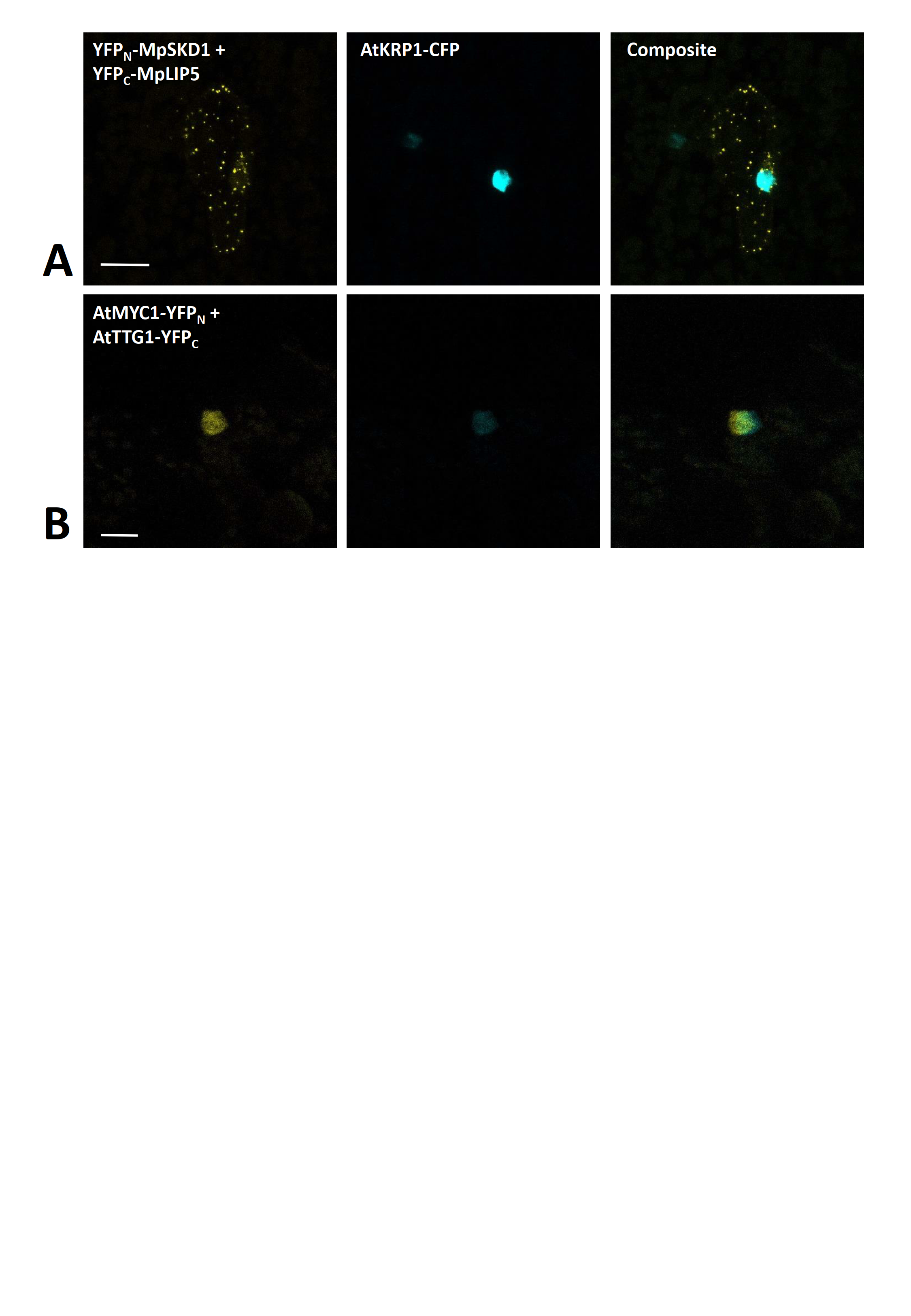

Supplement: Supplementary Figure 2 — Bimolecular fluorescent complementation assay quality controls. (A) The functionality of the negative control MpLIP5 was confirmed via co-bombardment of split-versions of MpLIP5 and the Marchantia homolog of the known Arabidopsis LIP5 interactor MpSKD1, showing a clear protein interaction in dot-like foci. (B) Split-YFP fusion constructs of AtMYC1 and AtTTG1, known interactors, were co-bombarded and shown to physically interact in M. polymorpha thallus epidermal cells, supporting the functionality of AtMYC1-YFPN. The constructs were co-bombarded with nuclear marker AtKRP1. Scale bar = 20 μm. Pictures show maximum projections of z-stack captions (see “Materials and Methods” section for details). [file Image_2.TIF]

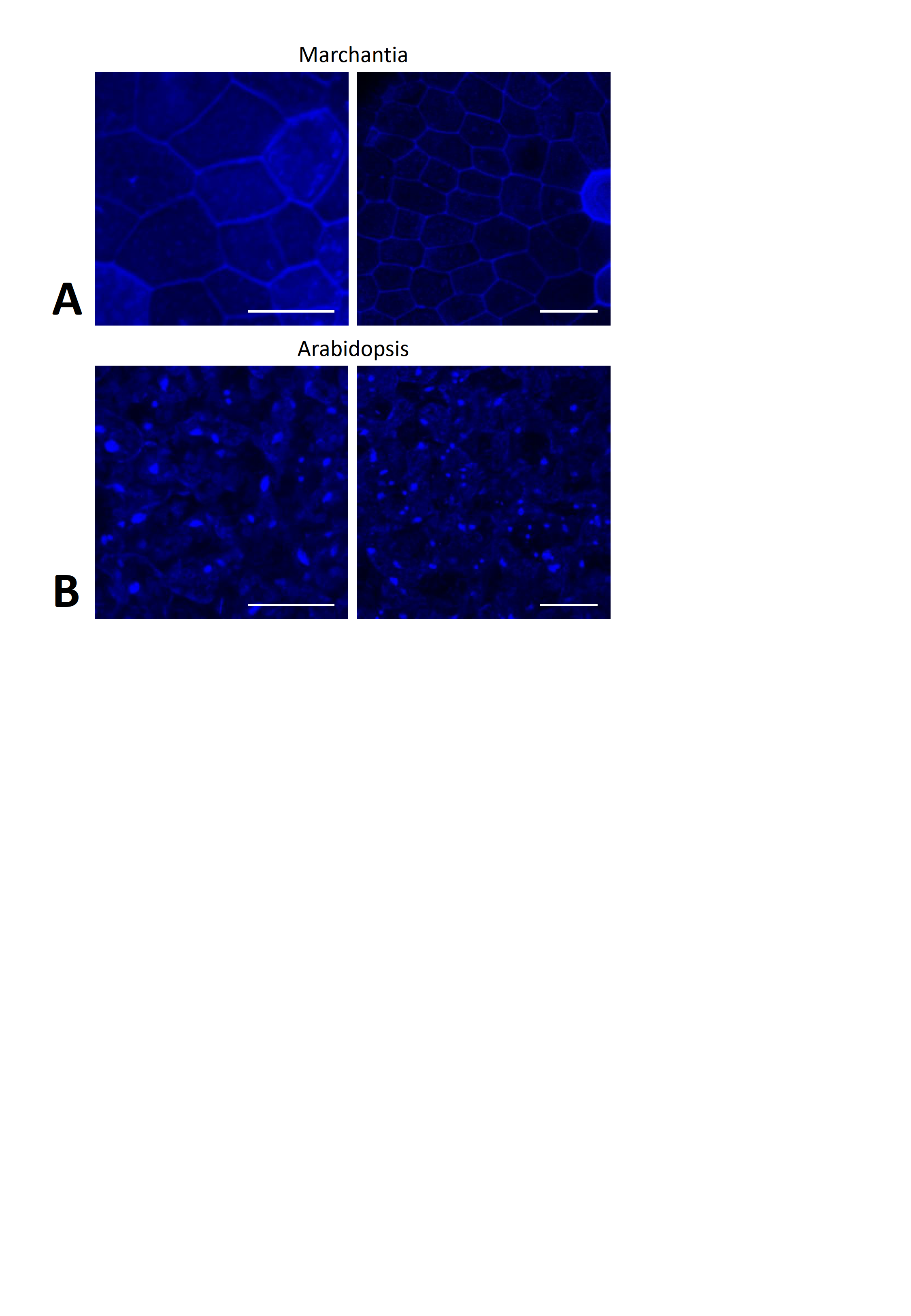

Supplement: Supplementary Figure 3 — Nuclei of M. polymorpha cannot be readily stained with DAPI. (A) DAPI staining of Tak-1 epidermal cells of a 4 days-old gemmaling. (B) DAPI staining of leaf epidermal cells of a 2 weeks old A. thaliana plant. Note the stained nuclei. All scale bars = 50 μm. [file Image_3.TIF]
