# Supplementary material for: A Comprehensive Toolkit for Quick and Easy Visualization of Marker Proteins, Protein–Protein Interactions and Cell Morphology in Marchantia polymorpha
Source: Front Plant Sci. 2020 Oct 15;11:569194. doi: 10.3389/fpls.2020.569194 (PMC7593560; doi:10.3389/fpls.2020.569194)
Supplement: Supplementary file 5 [file Data_Sheet_1.docx]

**Supplemental Table 1: Comprehensive list of all marker constructs used for biolistic transformation.** The list includes their origin (referenced publication or own generation), as well as the oligonucleotide sequences used as primers for amplification of new marker gene CDS. GW = Gateway-compatible cassette.

| **Construct** | **Generation / origin** | **Primers used for amplification of marker gene CDS** |
| --- | --- | --- |
| *proMpEF1α:: Lifeactin-Citrine* | Kimura and Kodama, 2016 | - |
| *pro35S::*AtAUN1-YFP | AtAUN1 CDS from Franck et al., 2018 remobilized into *pro35S*::GW-YFP | - |
| *pro35S::*AtAUN2-YFP | AtAUN2 CDS from Franck et al., 2018 remobilized into *pro35S*::GW-YFP | - |
| *pro35S*::AtDCP1-mCherry | Cloning of CDS in Steffens et al., 2015 remobilized into pAUBERGINE (M. Jakoby, GenBank ID: FR695418), kindly provided by A. Steffens | - |
| *pro*35S::AtKRP1-CFP | Cloning of CDS in Weinl et al., 2005 remobilized into pEXSG-CFP (Feys et al., 2005), kindly provided by M. Jakoby | - |
| *pro35S::*AtMRI-YFP | CDS from Boisson-Dernier et al., 2015 remobilized into *pro35S*::GW-YFP | - |
| *pro35S::*AtMYC1-YFP_N_ | Cloning of CDS in Pesch et al., 2013, remobilized into pSPYNE (Walter et al., 2004), kindly provided by L. Stephan | - |
| *pro*35S::AtNPSN12-mCherry | Cloning procedure as described in Steffens et al., 2014; CDS amplified with primers containing attB sites on pUNI clone U60291 recombined in pDONR207 and introduced into pAMARENA (M. Jakoby, GenBank ID: FR695418), kindly provided by M. Jakoby | - |
| *pro35S::*AtTTG1-YFP_C_ | Cloning of CDS in Bouyer et al., 2008, remobilized into pSPYCE (Walter et al., 2004), kindly provided by L. Stephan | - |
| *pro35S::*Citrine-mTalin | Kimura and Kodama, 2016 | - |
| *pro35S::*mCherry-AtDCP2 | Cloning of CDS in Steffens et al., 2015 remobilized into pAMARENA (M. Jakoby, GenBank ID: FR695418), kindly provided by A. Steffens | - |
| *pro35S*::mCherry-MpRAB5 | this study; CDS introduced into pAMARENA (M. Jakoby, GenBank ID: FR695418) | GGGGACAAGTTTGTACAAAAAAGCAGGCTCAATGGCCACCGCGGGAACGAA;  GGGGACCACTTTGTACAAGAAAGCTGGGTATCMGACGCAGCACATGCTTGATT |
| *pro35S*::YFP-MpRAB5 | this study; CDS introduced into pENSG-YFP (Feys et al., 2005) | GGGGACAAGTTTGTACAAAAAAGCAGGCTCAATGGCCACCGCGGGAACGAA;  GGGGACCACTTTGTACAAGAAAGCTGGGTATCMGACGCAGCACATGCTTGATT |
| *pro35S::*MpARA6-YFP | this study; CDS introduced into pEXSG-YFP (Feys et al., 2005) | GGGGACAAGTTTGTACAAAAAAGCAGGCTCAATGGGTTGTGCTGCCTCAGC;  GGGGACCACTTTGTACAAGAAAGCTGGGTATCMAGGCTTCTGGGTTGGCTGTC |
| *pro35S::*mCherry-SKL | SKL motif introduced into pAMARENA (M. Jakoby, GenBank ID: FR695418), kindly provided by M. Jakoby | - |
| *pro*35S::mCitrine-MpSYP13a | Kanazawa et al., 2015; received from Clement Champion, Prof. Liam Dolan Lab | - |
| *pro*35S::MpDCP1-YFP | this study; CDS introduced into pEXSG-YFP (Feys et al., 2005) | GGGGACAAGTTTGTACAAAAAAGCAGGCTCAATGGCACAAAATGGCAAGCCGATGC;  GGGGACCACTTTGTACAAGAAAGCTGGGTATCMTGTTGAATGTGCATTGAGCATCTCC |
| *pro*35S::MpDCP2-YFP | this study; CDS introduced into pEXSG-YFP (Feys et al., 2005) | GGGGACAAGTTTGTACAAAAAAGCAGGCTCAATGTCCGGCAACGCGCGTGC;  GGGGACCACTTTGTACAAGAAAGCTGGGTATCMGACTTCCAACTTTTGTATTATGCTT |
| *pro*35S::MpFER-YFP | this study; CDS containing GW-compatible attB1/2-sites, cloned into GW entry vector and introduced into *pro35S*::GW-YFP | GGGGACAAGTTTGTACAAAAAAGCAGGCTTAATGAGGCGTTCGTCTTGTTT;  GGGGACCACTTTGTACAAGAAAGCTGGGTTTAACCTTCCTTGAGGGTTCA |
| *pro*35S::MpMRI-YFP | MpMRI CDS from Westermann et al., 2019 remobilized into *pro35S*::GW-YFP | - |
| *pro35S*::YFP_C_-MpDCP1 | this study; CDS introduced into pCL113 (donated by J.F. Uhrig, unpublished data) | GGGGACAAGTTTGTACAAAAAAGCAGGCTCAATGGCACAAAATGGCAAGCCGATGC;  GGGGACCACTTTGTACAAGAAAGCTGGGTATCMTGTTGAATGTGCATTGAGCATCTCC |
| *pro35S*::*YFP_C_-*Mp*LIP5* | this study; CDS introduced into pCL113 (donated by J.F. Uhrig, unpublished data) | GGGGACAAGTTTGTACAAAAAAGCAGGCTCAATGGGGGAGACTGCGGATCCGAAGA;  GGGGACCACTTTGTACAAGAAAGCTGGGTATCMGTGAGCTTGTGATGAAGAAGAGGTC |
| *pro35S*::YFP_N_-MpDCP2 | this study; CDS introduced into pCL112 (donated by J.F. Uhrig, unpublished data) | GGGGACAAGTTTGTACAAAAAAGCAGGCTCAATGTCCGGCAACGCGCGTGC;  GGGGACCACTTTGTACAAGAAAGCTGGGTATCMGACTTCCAACTTTTGTATTATGCTT |
| *pro35S*::YFP_N_-MpSKD1 | this study; CDS introduced into pCL112 (donated by J.F. Uhrig, unpublished data) | GGGGACAAGTTTGTACAAAAAAGCAGGCTCAATGTACAGCAATTTCAAGGA  GGGGACCACTTTGTACAAGAAAGCTGGGTATCMACCCTCCTCACCAAATTCAC |
| *pUBQ10::*YFP*-*AtSYP32 / *pro35S*::CFP-AtSYP32 | Cloning procedure as described in Steffens et al., 2014; CDS amplified with primers containing attB sites on pUNI clone U20852, recombined in pDONR207 and introduced into pENSG-CFP/YFP (Feys et al., 2005), kindly provided by M. Jakoby | - |
| *pUBQ10::*YFP-AtGot1p homolog | Cloning procedure as described in Steffens et al., 2014; CDS amplified with primers containing attB sites on pUNI clone U63080 recombined in pDONR207 and introduced into  pENSG-YFP (Feys et al., 2005), kindly provided by M. Jakoby | - |

**Supplemental Table 2: Excitation and captured emission wavelengths used for analysis of fluorescent markers.**

| **Fluorophore** | **Excitation** | **Captured Emission** |
| --- | --- | --- |
| CFP | 458 nm | 470 nm – 480 nm |
| YFP | 514 nm/488nm | 524 nm – 530 nm |
| mCherry | 561 nm | 607 nm – 618 nm |
| FDA | 514 nm | 500 nm – 540 nm |
| PI | 561 nm | 610 nm – 630 nm |
| DAPI | 405 nm | 450 nm – 470 nm |
| FM4-64 | 514 nm | 600 nm – 640 nm |
| Hoechst33342 | 405 nm | 450 nm – 470 nm |

**Supplemental Table 3: Quantification of co-bombardment efficiency in *M. polymorpha* biolistic transformation.** Data from 9 independent co-transformation events of marker proteins used in this study. Scans of at least two transformed cells were used for the quantification.

| **Cells expressing both marker proteins** | **Total number of transformed cells** | **Efficiency of co-transformation [%]** |
| --- | --- | --- |
| 2 | 2 | 100 |
| 8 | 12 | 67 |
| 2 | 2 | 100 |
| 1 | 2 | 50 |
| 2 | 2 | 100 |
| 2 | 2 | 100 |
| 3 | 3 | 100 |
| 1 | 2 | 50 |
| 1 | 2 | 50 |
| 5 | 8 | 63 |
| 4 | 5 | 80 |
| 1 | 2 | 50 |
| 1 | 2 | 50 |
| 3 | 3 | 100 |
| 4 | 6 | 67 |
| 7 | 7 | 100 |
| 1 | 2 | 50 |
| 2 | 2 | 100 |
| 1 | 2 | 50 |
| 1 | 2 | 50 |
| 3 | 4 | 75 |
| 2 | 2 | 100 |
| 3 | 5 | 60 |
| **SUM: 60** | **SUM: 81** | **Average: 74** |
|  |  | SD: 23 |
